# Dr. Hamilton’s Surgical Dispensary

**Published:** 1847-04

**Authors:** Wm. H. White


ART. II.—*Dr. Hamilton's Surgical Dispensary.—Medical Department of the University of Buffalo.*—Reported by WM. H. WHITE.

The cases presented during the dissecting term are not noted below.

*Varicose Vims.*—Augustus Powers (a salt water tar,) aged 32.—Vena saphena interna of left leg varicose, caused by lifting at the capstan while on the coast of Africa. It first appeared about 10 years since, and burst two years since, producing a chronic varicose ulcer ; it bled copiously. It has several times been nearly healed, and again it has suddenly reopened, presenting a more extended surface than before.

Dr. H. operated by tying the vein about 2 or 3 inches below the patella, useing 3 ligatures ; two according to M. Reynaud's mode, and one a modification of Davat's mode. Two weeks after, patient appeared before the class and seemed highly pleased, he being able to walk quite well. There remains only two small ulcers which are rapidly closing.

*Conical Stump.*—Joseph Guild, aged 12, a native of Germany; about three years since, while there, he fell from a house and injured the right foot, and broke the bones of the leg. It was amputated, each bone being separated at the place of the fracture. The line of incision is still seen extending from the upper part of the lower fourth of the *tibia*, upwards and outwards to the lower part of the upper third of the *fibula*. The stump is conical and presents at its extremety an open ulcer.

Dr. H. reccommended a new amputation as the only means of cure.

*Chronic Rheumatism.*—Mrs. L. of Buffalo has had several attacks of acute rheumatism in the muscular and tendinous structure of the right arm.

Dr. H. prescribed tinct. of Colchicum ʒiv. tinct. guiac ʒi, sweet wine ℥iv. Dose, 2 tea spoons full, 3 times per day. Mrs. L. appeared three days after much improved. The arm being more flexible, and pain greatly diminished.

*Enlarged Tonsils, extirpated.*—Miss S., aged 12. The tonsils are much enlarged; have troubled her five years, causing deafness, and in the fall and winter slight attacks of quinsy.

Dr. H. removed them with Owen's instrument. Hæmorrhage slight.

*Displacement of Ulnar nerve.*—Mr. B., aged 30. On flexing the arm he feels a peculiar pain on the Ulnar side of the humerus, owing to a displacement of the Ulnar nerve. It can be replaced by the fingers, and then he can flex the arm without pain.

Dr. H. thought nothing but a bandage and rest could be of service. The patient did not feel willing to submit to either.

*Injury of Glutei Muscles.*—Mr. R., of Buffalo, slipped down some four years since, his legs being forceable abducted. Immediately afterwards he experienced an acute pain in the Glutei and Lumbar muscles.

Dr. H. prescribed a succession of blisters.

*Indirect Inguinal Hernia.*—Mr. M., aged 50. First noticed it about two years since; now as large as a butternut; external ring much enlarged.

Dr. H. advised Chase's truss.

*Chronic Inflammation of the Ligaments and Capsules of Ankle Joint.*—Inflammation of sub-acute character, slightly swollen. Dr. H. applied the *paste bandage.*

*Injury of the Chest.*—Mrs. P. Fell, injured the right pectoral muscles and pleura. The ribs were examined and found sound.—Prescribed stimulating linament.

*Fracture over superior longitudinal sinus.*—C. H., aged 12.—Was thrown from a horse about two months since, fractured left parietal bone. He was trephined by Dr. Wilcox of this City, and it healed kindly. The boy now appears well.

*Amaurosis.*—Mrs. M., aged 43. Amaurosis of right eye; sight impaired; has had inflammation of the eyes. Experiences an acute pain at the inner canthus, especially on touching the eye ball.

Dr. H. regards it as a case of functional Amaurosis from congestion. Prescribed a blister behind the ear, 2 leeches near the eye, and iv. gr. pill of blue-mass for four successive days, followed by cathartic.

*Foreign substance in the bronchial tubes.*—Miss G., aged 20.—About four months since, swallowed one side of a beech nut husk, which remained in left bronchial tube. She experiences much difficulty in breathing, and has occasionally severe attacks of coughing. Miss G. is healthy but plethoric. The whizzing sound produced at each inspiration and expiration is audible across the room. After a full examination, and consultation with eight or ten physicians and surgeons who were present, it was unanimously decided that no operation at present would be proper.

*Strabismus Internus of left eye.*—C. T., aged 20. He and his friends think it congenital. Left eye turned slightly inwards; the sight in this eye is not as good as in the other. Dr. H. operated with perfect success.

*Strabismus Internus of left eye.*—D. C., aged 8. Left eye turned towards inner canthus; the sight is affected; caused by inflammation. Dr. H. operated and the eye became straight.

*Irritable Ulcer.*—G. D., aged 50. Irritable ulcer on shin; round, and about the size of a dollar, with vertical and ragged edges. Dr. H. first prescribed bread and milk poultice, to be followed after a day or two by nitrate of silver in substance to the edges, adhesive straps, &c.

*Enlarged Tonsils extirpated.*—Miss M. A. G., aged 11. Enlarged Tonsils ; commenced 9 years since ; they have continued to increase; has quinsy occasionally, no hæmorrhagic tendency. Dr. H. extirpated them with Owen's instrument ; hæmorrhage very slight.

*Strabismus Convergens of both eyes.*—Mr J. aged 45. Originally both eyes were very much converged, operated on by Dr. H. about a year since. The fifth day after the opperation when he turned the right eye outwards, he could not see, which condition of matters lasted some days, and then disappeared. The parallelism of the eyes is now about half restored.

*Hydro-sarcocele.*—D. B. aged 28. Dr. H. drew off about a pint of water and reccommended Hyd. Iod. pot. ung. for enlarged testicle.

*Indolent Ulcer and Ectropion of left upper lid.*—H. W. aged 29.—Caused by syphilis, contracted two years since. Has an indolent ulcer on right leg twice the size of a dollar ; has had several other ulcers on the face and different parts of the body, also Ectropion of upper lid.

Dr. H. advised nitrated merc. ung. for ulcer, and for the lid he reccommended a plastic operation.

*Strabismus Internus of left eye.*—H. W. aged 16. Left eye turned *slightly* towards the inner canthus. This eye he cannot open quite as far as the other. He was four years old when it occurred, caused by hooping cough.

Dr. H. advised him not to submit to an operation, but to use it by looking steadily at a small black object, with the right eye at first closed, and then to open the right eye suddenly and look with both.

*Chronic Eczema.*—G. R. B. aged 24. Commenced 20 years since; first appeared on the shoulder resembling a ring worm ; not hereditary ; occasionally some enlargement in the groin. The eruption came out 8 years since to a great extent, he then visited Avon springs and remained four months. For a time the waters helped him.—He has been costive as long as he can remember. The eruption covers the whole body. He took for a long time Vaughn's Lithontriptic, and as long as the cathartic effect was kept up it benefited him. This is the case of "Leprosy" which Vaughn announces cured.

Dr. H. advised a bath of sup. carb. sodæ, and a gentle cathartic compound of rhubarb, aloes and magnesia. The prognosis is doubtful.

